# Supplementary material for: Nano scale instance-based learning using non-specific hybridization of DNA sequences
Source: Commun Eng. 2023 Dec 8;2:87. doi: 10.1038/s44172-023-00134-8 (PMC10955820; doi:10.1038/s44172-023-00134-8)
Supplement: Supplementary file 2 — Supplementary Information [file 44172_2023_134_MOESM2_ESM.pdf]

Supplementary Notes

for

Nano scale instance-based learning using non-specific hybridization of DNA sequences

Yanqing Su, Wanmin Lin, Ling Chu, Xiangzhen Zan, Peng Xu, Fengyue Zhang, Bo Liu, Wenbin Liu

Supplementary Note 1. Hybridization of DNA moleculars.

Hybridization is a phenomenon in molecular biology whereby a ssDNA can anneal to another ssDNA that follows Watson-Crick base pairing (A with T and C with G). The hybridization process of DNA molecules has several key characteristics:

1) Specificity. A ssDNA prefers to hybridize with the perfectly reverse complement one (specific hybridization, Supplementary Figure 1a)

2) Non-specificity. In the absence of perfectly reverse complement in a given system, a ssDNA may hybridize with a partial reverse complement one (non-specific hybridization, Supplementary Figure 1b and 1c). This phenomenon can make it challenging to precisely predict yield by edit distance.

3) Parallelism.Hybridization between ssDNA occurs simultaneously in a system which is a good fit for massively parallel DNA data processing.

4) Temperature sensitivity. The sturctures of dsDNA is notably impacted by tempture. (Supplementary Figure 1d) At elevated temperatures, dsDNA may dissociate into ssDNA. While lowering the tempture allows the complementary strands to reassociate and form a hybrid. This temperature sensitivity ensures the operability of hybridization control in wet-lab: raise the tempeture of the system to denature dsDNA or secondary knots of ssDNA when prepare the system for classification. Slowly cool the system to obtain dsDNA and acquire classification results

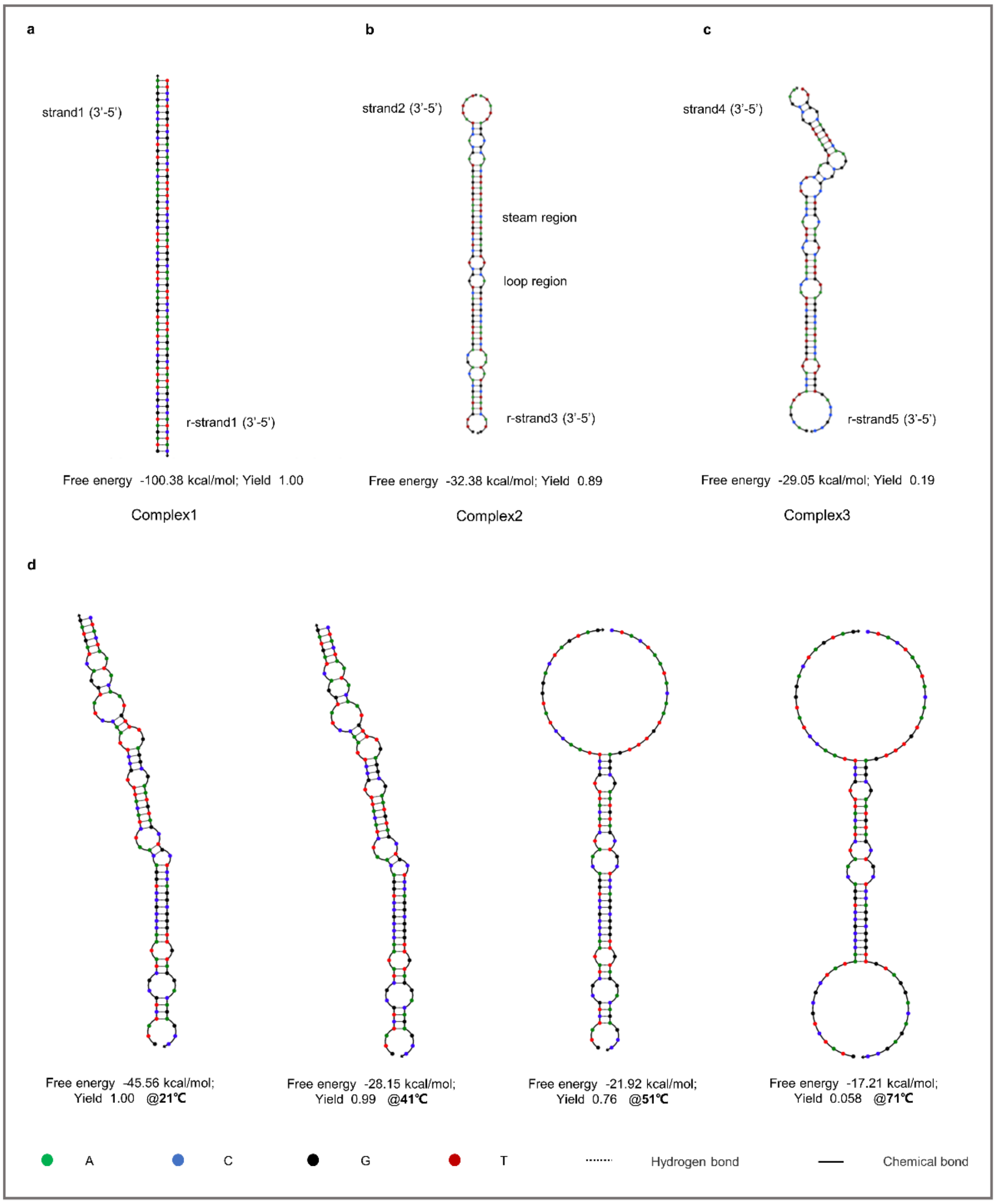

Supplementary Figure1 Visualization of hybridization between DNA sequences. r-strand* represents reverse complement of strand*. **a.** Specific hybridization of DNA sequences. **b & c.** Non-specific hybridization of DNA sequences. Edit distances between strand2 and strand3, strand4 and strand5 are the same, but complex2 and 3 have very different free energy and yield. The stem region is a sub-sequence that reversely complemented. The loop region is a mis-match area. **d.** Different structures, yield and free energy of a given dsDNA in different temperatures. As the temperature increases, the stability of dsDNA become compromised. All structures, free energy, and yield in the fig are reported by NUPACK.

Supplementary Note 2. Loss function of the encoder.

The flowchart of encoding loss and sequence loss is depicted in Supplementary Figure 3. For the ease of programming implementation, we punish the ambiguous

positions by generating target for the encoder. (Supplementary Figure 2a) For the sequence loss, similarity of the feature vector is measured by T_1=16 firstly. (Figure2c, image pairs may have the same label or visually similar when Euclidean distance of corresponding feature vectors smaller than 16.) Second, the yield. We draw Buterez, D *et al.*'s experience[25] to set T_2=0.8. A dsDNA complex with yield larger than 0.8 is considered to be a stable structure. (Supplementary Figure 1). Finally, the MNIST labels. Some paths (green in Supplementary Figure 2b) skip the judgement of labels to avoid excessively server punishment.

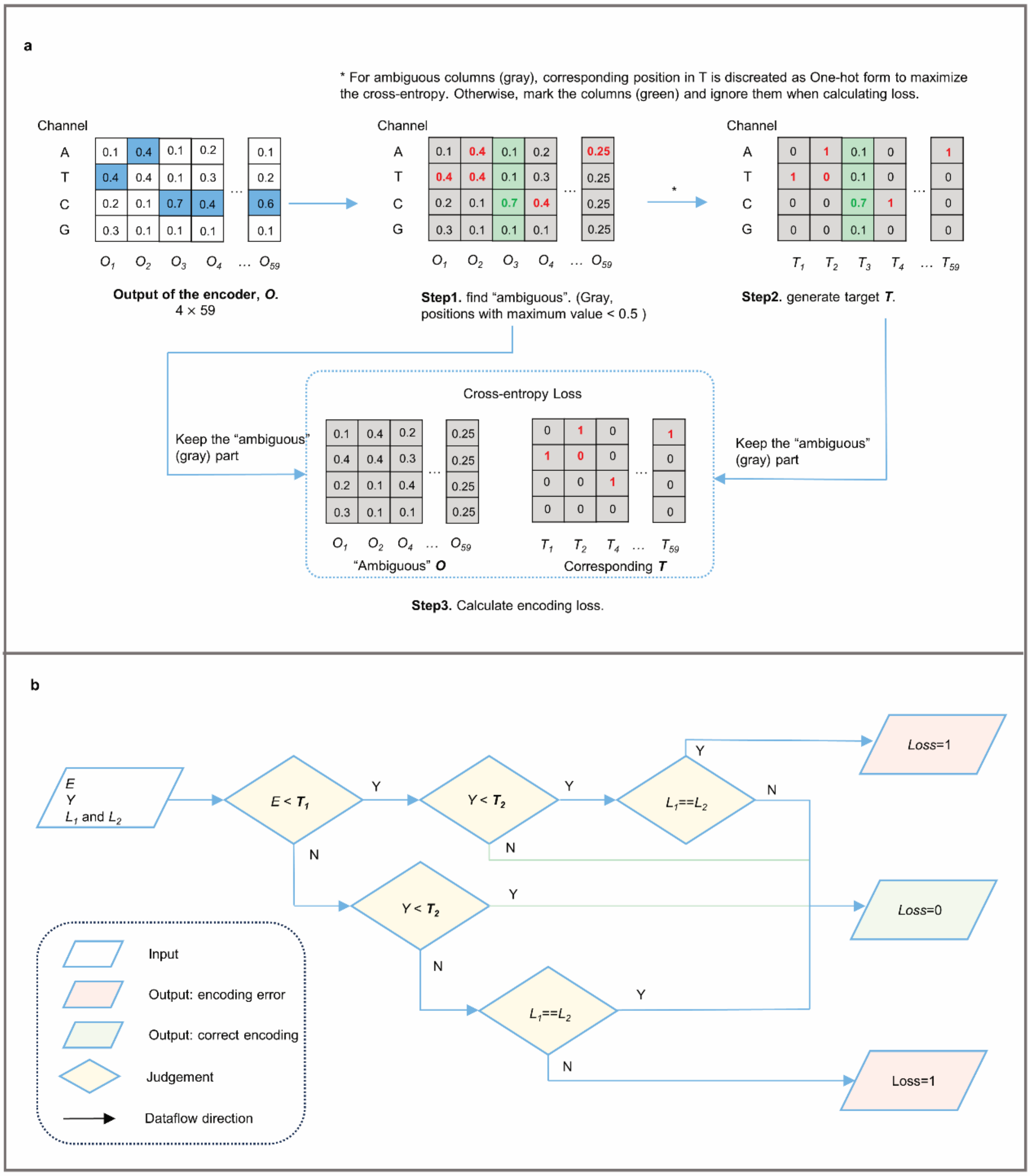

Supplementary Figure2 Flowchart of the encoder loss function. a. Steps of

calculating encoding loss. Target is generated according to the output of the encoder. **b.** Flowchart of calculating sequence loss. Sequence loss has three inputs: the Euclidean distance of encoded image feature vectors (E), the yield of encoded DNA reported by the predictor (Y) and MNIST labels (L_1 and L_2) of encoded images. T_1 and T_2 are different hyper-perimeters. Lines in green indicate paths skip the judgement of labels. The loss function has binary output: 1 for encoding error and 0 otherwise.

Supplementary Note 3. Options of NUPACK. NUPACK is an authoritative software suite for the analysis of DNA, which has some optional parameters can be defined by the user. Such as:

1) Temperature. Since hybridization of DNA sequences is temperature-sensitive, NUPACK requires a declaration of temperature or temperature range.
2) Model options. Analysis method, hyper-parameters set and free energy parameter set for NUPACK. See https://www.nupack.org/ for more information.
3) Species. Specify DNA sequences to analysis and their initial concentration.
4) Max complex size: NUPACK automatically analysis all complexes up to the specified maximum number of strands.

Supplementary Note 4. Selected fuzzy query sequences in hard-to-classifiy situation.

**MNIST label**	**Query sequence**<br>(Length:59nt. From 5' to 3')
0	TTACCGGTGCAAGGATTGAGTCCAATTGATATGCTCCAGTCGGGTTATCTGGCGGCGAC
1	CTGCCACATGACTCGTATGATGAGCTAGTGACATGGGTCTTGGGACGACTAGCAAATAG
2	GTGTTTGTGAGGTTGGCTCGGCGGGATTTATTCGGTAGGCGTAGGAGTGTCCACGCTTG
3	GTGAGTGTAGGGTTGGCTAGGTGAGATTAATTAATTGGTTGTGACCTTGTCCATTCTTA
4	ATAATAATTTATGTTTTGTTTTTTAGTAGAAAATAATATCTTTTTTCTTATTTTATTGA
5	TCATTTCGAAGTAGCTGGGTAGTCCAAGTCGTTTTGTGTCGCTTCGCGAACTCCTCGCG
6	ATTTCTCCCCTGTTGACGCATAATGTCTACTGGTTATTGTCTCTCCCTGCGTCCGAAAT
7	TTTTGCCGATCGTCCATAATCGCAGGGTGCGAACCGAAGGGATTTCTATCTTGTGCGTT
8	AAGTTTTCAATAAAAGGTGAAGGCGAAGGTGCAATGGTGGAAGGTACAGGGGTAAAGCG
9	TAATCTTTGTATAGGTCAGCCTAGCGCTCGCCGATAATTGTATATGGAATCCGTTCCTA

Supplementary Table 1. Query sequences corresponding to images in Figure 3a.

Supplementary Note 5. Detail analysis of misclassified imags in dry-lab.

Supplementary Table 2 shows the record of 51 misclassified samples in dry-lab experiment. Some of them are similar to other numbers ((II, E) and (IV, F) especially), some are blurry (class 8 in Figure 3d). For the first case, the query may fall into other clusters leading to classification errors (maximum yield sum > 2000, such as (I, A), (I, B) and (I, C)). For the second case, the query may be an "isolated" point and surrounded by less labeled sequences (maximum yield < 1000, such as (I, E), (I, F) and (I, H)), which leads a classification failure as well.

ID	1	2	3	4	5	6	7	8	9	10
(Ⅰ, A)	(8, 3231)	(6, 2212)	**(0, 1434)**	(9, 521)	(5, 87)	(1, 54)	(2, 32)	(3, 25)	(4, 17)	(7, 15)
(Ⅰ, B)	(6, 3768)	(8, 2469)	(4, 2275)	***(0, 2127)***	(9, 1459)	(1, 985)	(2, 54)	(7, 16)	(3, 14)	(5, 11)
(Ⅰ, C)	(6, 2243)	***(0, 2168)***	(5, 1954)	(8, 1137)	(9, 454)	(7, 121)	(2, 84)	(1, 63)	(4, 51)	(3, 0)
(Ⅰ, D)	(6, 3174)	***(0, 2957)***	(8, 1213)	(5, 63)	(9, 31)	(2, 24)	(7, 22)	(1, 15)	(3, 10)	(4, 8)
(Ⅰ, E)	(2, 687)	***(1, 343)***	(7, 219)	(6, 154)	(9, 121)	(8, 64)	(0, 53)	(3, 44)	(4, 30)	(5, 22)
(Ⅰ, F)	(7, 989)	(4, 636)	***(1, 298)***	(6, 254)	(9, 232)	(2, 61)	(8, 58)	(3, 37)	(0, 30)	(5, 0)
(Ⅰ, G)	(7, 2389)	***(2, 2205)***	(1, 1176)	(3, 909)	(4, 724)	(5, 91)	(9, 18)	(6, 17)	(0, 12)	(8, 1)
(Ⅰ, H)	(4, 582)	(3, 447)	***(2, 261)***	(8, 52)	(9, 30)	(7, 25)	(6, 25)	(1, 14)	(5, 13)	(0, 6)
(Ⅰ, I)	(0, 3439)	***(2, 2189)***	(7, 854)	(3, 189)	(9, 187)	(8, 98)	(6, 64)	(1, 55)	(4, 5)	(5, 1)
(Ⅰ, J)	(3, 3561)	***(2, 915)***	(7, 328)	(4, 162)	(0, 94)	(1, 61)	(9, 55)	(6, 24)	(8, 10)	(5, 0)
(Ⅰ, K)	(7, 1952)	***(3, 1763)***	(8, 143)	(5, 158)	(4, 99)	(1, 73)	(2, 64)	(0, 51)	(9, 32)	(6, 31)
(Ⅱ, A)	(7, 1553)	(0, 695)	(5, 34)	***(3, 21)***	(4, 17)	(9, 12)	(2, 12)	(1, 10)	(8, 9)	(6, 0)
(Ⅱ, B)	(0, 543)	***(3, 325)***	(8, 51)	(4, 25)	(9, 23)	(5, 21)	(2, 19)	(1, 17)	(7, 10)	(6, 8)
(Ⅱ, C)	(7, 879)	***(3, 619)***	(8, 351)	(9, 37)	(6, 24)	(2, 19)	(1, 17)	(5, 13)	(4, 12)	(0, 10)
(Ⅱ, D)	(9, 985)	(1, 645)	***(4, 198)***	(6, 164)	(5, 89)	(0, 32)	(3, 19)	(2, 14)	(7, 0)	(8, 0)
(Ⅱ, E)	(9, 4760)	(7, 4230)	(8, 1589)	***(4, 1236)***	(3, 1103)	(5, 1007)	(1, 942)	(0, 936)	(2, 915)	(6, 837)
(Ⅱ, F)	(9, 3537)	(3, 2852)	***(4, 2652)***	(8, 558)	(7, 524)	(2, 363)	(0, 321)	(5, 169)	(1, 105)	(6, 69)
(Ⅱ, G)	(9, 2551)	***(4, 2469)***	(7, 189)	(6, 186)	(8, 128)	(0, 121)	(5, 107)	(1, 43)	(2, 23)	(3, 0)
(Ⅱ, H)	(9, 3881)	(7, 1661)	***(4, 587)***	(5, 128)	(1, 103)	(2, 96)	(0, 86)	(3, 59)	(8, 50)	(7, 42)
(Ⅱ, I)	(7, 448)	(9, 205)	(3, 62)	(5, 50)	***(4, 37)***	(1, 16)	(2, 16)	(0, 9)	(6, 7)	(8, 7)
(Ⅱ, J)	(7, 886)	(1, 696)	(9, 120)	***(4, 108)***	(0, 98)	(8, 97)	(2, 65)	(5, 56)	(3, 24)	(6, 16)
(Ⅱ, K)	(7, 528)	***(4, 501)***	(9, 137)	(0, 96)	(3, 62)	(1, 15)	(6, 13)	(2, 5)	(8, 4)	(5, 3)
(Ⅲ, A)	(9, 654)	***(4, 636)***	(7, 501)	(1, 28)	(6, 14)	(3, 2)	(2, 1)	(0, 1)	(5, 1)	(8, 1)
(Ⅲ, B)	(7, 756)	***(4, 701)***	(9, 236)	(1, 63)	(6, 54)	(8, 12)	(3, 7)	(2, 5)	(5, 5)	(0, 2)
(Ⅲ, C)	(1, 278)	***(4, 259)***	(7, 236)	(9, 184)	(6, 86)	(3, 57)	(8, 41)	(5, 33)	(0, 29)	(2, 26)
(Ⅲ, D)	(7, 3924)	***(4, 3561)***	(9, 1875)	(1, 989)	(6, 652)	(3, 533)	(5, 485)	(8, 431)	(2, 418)	(0, 7)
(Ⅲ, E)	(7, 443)	(9, 337)	***(4, 312)***	(6, 107)	(2, 36)	(1, 33)	(5, 24)	(3, 15)	(0, 12)	(8, 6)
(Ⅲ, F)	(9, 518)	***(4, 494)***	(7, 263)	(0, 216)	(3, 144)	(2, 97)	(8, 86)	(1, 70)	(6, 56)	(5, 12)
(Ⅲ, G)	(3, 4105)	***(5, 3938)***	(6, 1957)	(0, 337)	(2, 201)	(1, 104)	(8, 96)	(7, 94)	(4, 21)	(2, 0)
(Ⅲ, H)	(7, 303)	(3, 285)	***(5, 254)***	(4, 101)	(0, 96)	(1, 57)	(2, 41)	(8, 35)	(6, 31)	(9, 28)
(Ⅲ, I)	(3, 2936)	(6, 2781)	***(5, 2563)***	(8, 364)	(9, 145)	(7, 32)	(1, 25)	(2, 21)	(0, 7)	(4, 6)
(Ⅲ, J)	(3, 3108)	***(5, 3074)***	(6, 2503)	(7, 415)	(4, 173)	(2, 128)	(0, 121)	(9, 117)	(8, 108)	(1, 104)
(Ⅲ, K)	(3, 523)	***(5, 401)***	(6, 102)	(8, 46)	(7, 21)	(1, 20)	(0, 17)	(9, 15)	(2, 15)	(4, 13)
(Ⅳ, A)	(3, 2754)	***(5, 2696)***	(6, 149)	(2, 121)	(8, 109)	(0, 98)	(1, 45)	(4, 19)	(9, 12)	(7, 6)
(Ⅳ, B)	(2, 3156)	(8, 2965)	***(5, 2857)***	(3, 2505)	(6, 2143)	(0, 1958)	(7, 734)	(1, 658)	(9, 242)	(4, 164)
(Ⅳ, C)	(6, 698)	***(5, 451)***	(0, 192)	(3, 99)	(8, 46)	(9, 17)	(4, 15)	(1, 12)	(7, 9)	(2, 0)
(Ⅳ, D)	(4, 2749)	(9, 2657)	(7, 2431)	***(6, 2408)***	(8, 1505)	(0, 741)	(1, 582)	(5, 301)	(3, 257)	(2, 239)
(Ⅳ, E)	(7, 2569)	***(6, 2431)***	(4, 1230)	(9, 698)	(0, 582)	(1, 189)	(2, 74)	(3, 51)	(8, 13)	(5, 11)
(Ⅳ, F)	(4, 3014)	***(6, 2958)***	(9, 1808)	(7, 843)	(1, 564)	(0, 251)	(3, 113)	(8, 63)	(5, 13)	(2, 5)
(Ⅳ, G)	(2, 1912)	(1, 1837)	***(7, 1634)***	(8, 925)	(5, 491)	(3, 228)	(4, 61)	(0, 25)	(6, 22)	(9, 0)
(Ⅳ, H)	(8, 941)	***(7, 926)***	(1, 459)	(9, 431)	(4, 98)	(2, 54)	(3, 17)	(0, 12)	(5, 6)	(6, 5)
(Ⅳ, I)	(1, 1398)	***(7, 1293)***	(9, 321)	(8, 196)	(4, 193)	(6, 65)	(0, 28)	(2, 26)	(3, 24)	(5, 21)
(Ⅳ, J)	(1, 1132)	(2, 1058)	***(7, 891)***	(3, 64)	(6, 51)	(0, 47)	(8, 6)	(4, 6)	(5, 3)	(9, 0)
(Ⅳ, K)	(1, 1689)	***(7, 1433)***	(9, 1368)	(2, 1265)	(8, 870)	(3, 566)	(0, 538)	(4, 489)	(6, 476)	(5, 424)
(Ⅴ, A)	(9, 1081)	(1, 1008)	***(7, 898)***	(8, 827)	(3, 331)	(2, 325)	(6, 109)	(5, 84)	(0, 28)	(4, 27)
(Ⅴ, B)	(1, 756)	***(7, 703)***	(9, 504)	(2, 215)	(8, 194)	(6, 93)	(4, 91)	(0, 33)	(3, 16)	(5, 9)
(Ⅴ, C)	(6, 501)	***(8, 482)***	(4, 107)	(2, 89)	(3, 86)	(9, 25)	(0, 23)	(1, 11)	(5, 6)	(7, 2)
(Ⅴ, D)	(9, 692)	***(8, 690)***	(3, 325)	(7, 264)	(0, 172)	(2, 89)	(1, 47)	(4, 22)	(6, 21)	(5, 17)
(Ⅴ, E)	(4, 641)	***(9, 505)***	(7, 298)	(8, 234)	(3, 221)	(2, 79)	(1, 32)	(5, 31)	(0, 14)	(6, 9)
(Ⅴ, F)	(0, 1236)	(7, 1158)	***(9, 1069)***	(2, 834)	(6, 341)	(5, 124)	(4, 97)	(1, 96)	(3, 39)	(8, 4)
(Ⅴ, G)	(0, 2651)	(8, 2105)	***(9, 2058)***	(7, 1254)	(6, 581)	(3, 294)	(2, 185)	(1, 174)	(4, 156)	(5, 143)

Supplementary Table 2. **The record of misclassified samples.** Column ID: pairs (row, column) locate misclassified images in Figure 3d. Column 1 to 10: yield records of each misclassified image (sorted by yield sum). Data are presented as pairs (class, yield sum between the query and all training samples of the class). Bold italic one indicates the ground truth class (by MNIST dataset) of this misclassified image.
